# Current status of hair analysis in forensic toxicology in China

**DOI:** 10.1080/20961790.2021.1921945

**Published:** 2021-07-09

**Authors:** Hui Yan, Ping Xiang, Min Shen

**Affiliations:** Department of Forensic Toxicology, Academy of Forensic Science, Shanghai Key Laboratory of Forensic Medicine, Shanghai, China

**Keywords:** Forensic sciences, forensic toxicology, hair analysis, black hair, drug abuse, microsampling, imaging mass spectrometry

## Abstract

Hair analysis has been mainly used to document drug use history in abusers, drug-facilitated crime cases, doping control analysis and postmortem toxicology in the fields of forensic toxicology, clinical toxicology, and doping control. Hair analysis has also gained more attention in the last 30 years in China. Relevant technology has been promoted as more research has appeared concerning hair analysis, and consensus has been sought among forensic toxicologists regarding aspects such as hair decontamination treatment, detection of abused substances in hair, segmental hair analysis and interpretation of analytical results. However, there are still some limitations in the estimation of drug intake time and frequency by segmental hair analysis due to the different growth cycles evident within a bundle of hairs, the drug incorporation mechanism and sampling errors. Microsampling and imaging mass spectrometry (IMS) technology based on a single hair may be a good choice to estimate drug intake time more accurately. Analysis of hair root samples may also be used to document acute poisoning in postmortem toxicology, and the analysis of the hair shaft can document long-term use of drugs depending on the length of the hair being evaluated.

## Introduction

It is one of the important missions in forensic toxi­cology to search for scientific biological matrices of great evidential value that can provide detailed information. Taking advantage of target stability, a long detection window and the information about the long-term history, hair analysis has gained more attention in forensic toxicology, clinical toxicology, and doping control in the last 30 years. Internationally renowned forensic toxicologists such as Kintz, Musshoff and Drummer have promoted hair analysis technology and applications with their research results in hair decontamination treatment, detection of abused substances in hair, segmental hair analysis and interpretation of analytical results [1–6]. In 2016, Armitage and Rogers [7], arguing that “sophisticated analytical techniques are giving hair a new role in forensics”, affirmed the evidential value and research value of hair.

In China, hair analysis went through a similar process. The treatment of hair, gas chromatography-mass spectrometer (GC-MS) and liquid chromatography tandem-mass spectrometry (LC-MS/MS) analysis were developed and optimized in scientific research. During the past 30 years, the detection of anabolic steroids in black hair, the drug concentration-time relationship in hair and a kine­tics model were investigated; also, the identification and evaluation system of psychoactive substances in hair was established [8–20]. Then, information related to whether the subject took the drug or not, the type of drug and the drug ingestion history were provided in casework in judicial practice. Hair ana­lysis in China is now used not only for handling drug addiction and rehabilitation cases but also for drug-facilitated crime (DFC) cases, such as drug-facilitated sexual assault, robbery and fraud, to provide evidence of drug use at the time of the crime, as well as in postmortem toxicology.

## Current application of hair analysis in China

### Confirmation of drug intake and investigation of drug use history

Compared with biological samples such as blood and urine, hair analysis has the advantages of stable presence of exogenous drugs, extended detection window, and the ability to reflect long-term drug abuse history. In 1999, meperidine was detected in a 22-cm-long hair from a 34-year-old female by segmental hair analysis, and the history of meperi­dine abuse was verified by the distribution of meperidine in the hair shaft [21]. In 2000, a small amount of monoacetylmorphine and morphine was detected in the hair of an 18-month-old baby 2 weeks after a negative urine test, indicating that he had suffered retaliatory heroin injections [22]. In 2009, methamphetamine and amphetamine were detected in drug abusers; detected amounts ranged from 0.24–33.80 ng/mg and 0.22–1.52 ng/mg, respectively [23]. In recent years, the appearance of new psychoactive substances (NPSs) has posed significant public health issues comparable to those of drugs abused by older individuals. Methods have been established for the determination of fentanyls, cathinones, tryptamines and their metabolites in human hair by LC-MS/MS and liquid chromatography-high-resolution mass spectrometry (LC-HRMS) [24–26]. The ratio of amphetamine-to-methamphetamine concentration in hair from methamphetamine abusers was 0.015–0.384 (*n* = 104) [27]. 5-MeO-DIPT was identified in human hair with amounts ranging from 0.1 to 13 000 pg/mg (*n* = 77) [24, 28].

Animal models, volunteer experiments, and hair samples from actual cases were used to study the relationship between drug concentrations found in hair and the dose of drug intake, the effect of hair colour, the confirmation of the drug use, drug use history, and the way the drug was introduced into the body.

The relationship between the physicochemical properties of the drug and the amount of the drug that infiltrates the hair

Studies have found that the speed and extent of drugs entering black hair are related to the molecu­lar weight, polarity and lipid solubility of the drugs. The amount of psychotropic drug that infiltrates the hair after administration of the same dose is roughly in the following order: carbamazepine > antan > amitriptyline > doxepin > haloperidol > trifluoperazine > chlorprothixene > chlorproma­zine > clozapine [29]. The research results also showed that there are relatively few metabolites in hair, and they are usually the most easily formed in the body, with strong lipophilicity and relatively high content. For example, six metabolites are detected in the urine of meperidine abusers, whereas only relatively high levels of normeperidine, N-hydroxymethyl meperidine and N-acetyl meperidine are detected in the hair of meperidine abusers [8, 30]. The identification of drugs and their metabolites in hair can provide indisputable evidence of drug intake.

The relationship between drug concentration in hair and the dose of drug intake

Volunteer experiments and actual case study results show that the concentrations of more than 20 drugs in black hair are positively correlated with the doses of the drugs used. For example, there was a positive correlation between chlorpromazine and clozapine concentrations in the hair of patients, and the drug dose in treatments yielded *R* = 0.8047 (P < 0.001, *n* = 16) and *R* = 0.7097 (P < 0.001, *n* = 16) [29]. However, there are also some drugs, such as aripiprazole and risperidone, that do not have a signifi­cant correlation with the dose and the concentration in the hair [31]. Establishing a model linking the dose-concentration relationship requires a large amount of data and the consideration of individual differences. Although it is not feasible to infer specific drug doses based on the drug concentration in the hair, the drug concentration in the hair can still provide information on the intensity and frequency of the drug, helping to explain the analytical results.

The effect of hair colour on hair drug concentration

The experiments of guinea pig models with different hair colours showed that the drug concentration in hair is obviously related to hair colour; that is, the drug concentration in hair is black > brown > white. The average ketamine concentration ratio of black to brown hair is 1.87, and that of black to white hair is 2.24 [31]. The concentration of ketamine in brown hair tends to be consistent with that in black hair after correction for the melanin content in hair (7.11 μg/mg for black hair and 3.57 μg/mg for brown hair), which was suggested by Rollins et al. [32]. This result verifies the hypothesis that the drug mainly binds to melanin in hair [29].

The distribution and elimination characteristics of drugs in black hair

**Figure 1.**
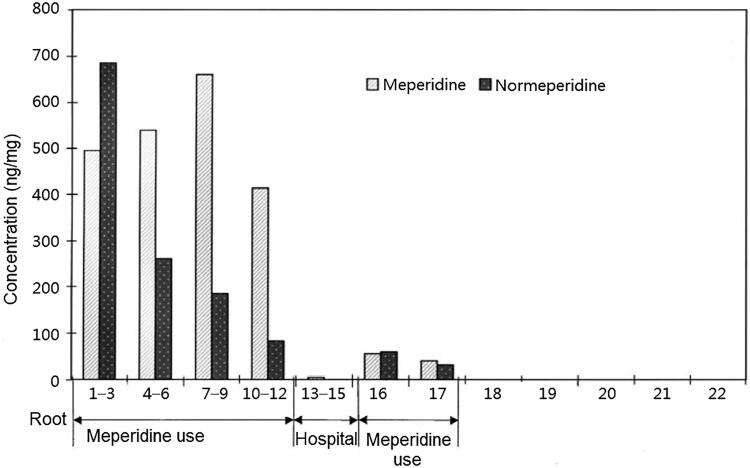
Meperidine distribution in hair and meperidine use history. Adapted with permission [21].

The concentration-time course of more than 20 drugs, such as ketamine, clonazepam, and anabolic steroid, in the hair shaft was investigated by segmental hair analysis. The results showed that the distribution of most drugs in the hair shaft was related to a history of abuse. For example, a change in meperidine concentration in hair could be explained by the pattern of meperidine ingestion. In one of our research in 1999 [21], high meperidine and normeperidine levels in 1–12 cm hair segments corresponding to the last 10 months were evident in one case heavily addicted to meperidine, trace meperidine in 13–15 cm hair segments corresponded to 3 months of hospital time, a low concentration of meperidine and normeperidine in 16–17 cm hair segments corresponded to a 2-month meperidine use history prior to hospitalization. In this way, segmental hair analysis could indicate that meperidine was abused again after a short period of hospital rehabilitation (Figure 1) [21]. Another example is that by studying the elimination kinetics of monoacetylmorphine, morphine, codeine, methamphe­tamine and amphetamine in hair, the corresponding elimination curve, elimination half-life and elimination rate constant were obtained [14]. According to the hair growth rate of approximately 1 cm/month, the elimination half-lives of monoacetylmorphine, morphine and codeine in hair were 0.88 months (95%CI, 0.74–1.03), 0.73 months (95%CI, 0.64–0.81), and 0.61 months (95%CI, 0.54–0.69), respectively[14]. The elimination half-lives of S-methamphetamine, R-methamphetamine, S-amphetamine and R-amphetamine on the hair shaft were 0.64 (95%CI, 0.46–0.96), 0.58 (95%CI, 0.41–0.93), 0.62 (95%CI, 0.49–0.88) and 0.50 months (95%CI, 0.42–0.56), respectively [33]. Therefore, when hair analysis is used to confirm the history of drug ingestion, according to the current prescribed threshold and the 0–3 cm hair segments (from hair root) used, if the suspect has a history of drug abuse, there should be no less than a 6-month interval between hair sampling and the last penalized use of illegal drugs. Then, false positive results caused by past drug use can be avoided.

On the basis of domestic and international drug abuse research results and application practices, Measures for the Identification of Drug Addiction (http://www.gov.cn/gongbao/content/2011/content_1913190.htm) were implemented in China. In 2017, the detection of drugs in human hair samples was included as evidence for the identification of drug addicts. In 2018, the Specifications for the Detection of Drugs in Human Hair Samples (https://www.scxsls.com/knowledge/detail?id=143343) were formulated, which stipulated the specific requirements for the extraction, storage, submission and testing of hair samples, as well as the threshold (cut-off value) for laboratory hair testing (Table 1). The threshold is consistent with the recommendations of the Society of Hair Testing (SoHT) [35], ensuring comparability between different labs and consistency with international rules. The authors’ laboratory conducted a proficiency test of Analysis of Abuse Substances in Hair (http://www.ssfjd.com/CNAS/Default.aspx) in China to ensure the hair analysis capabilities of forensic toxicology laboratories.

**Table 1.** Cutoff value included in specifications for the detection of drugs in human hair samples.

Group	Drug	Cutoff (ng/mg)
Opiates	6-Acetylmorphine (6-MAM)	0.20
	Morphine	0.20
Amphetamines	Amphetamine	0.20
	Methamphetamine	0.20
	MDMA	0.20
	MDA	0.20
Cathinones	Methcathinone	0.20
Ketamine	Ketamine	0.20
	Norketamine	0.20
Cocaine	Cocaine	0.50
	Benzoylecogonine	0.05
Cannabinoids	THC	0.05

**Figure 2.**
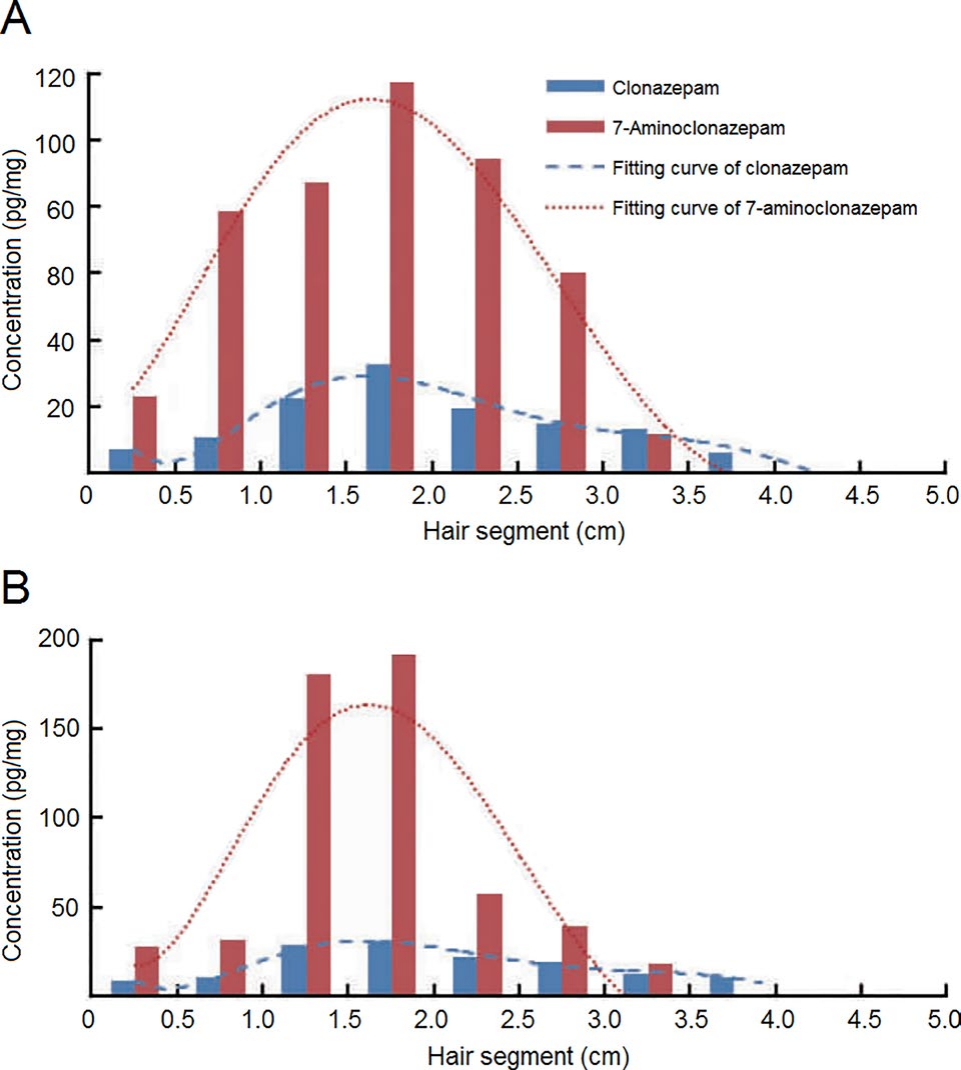
Distribution of clonazepam and its metabolite 7-aminoclonazepam in the hair of drug-facilitated crime (DFC) victims (A) Victim 1. (B) Victim 2. Adapted with permission [41].

The implementation of specifications has greatly furthered the progress and application of hair ana­lysis technology in China. In Shanghai, in 2018, 14 184 cases requested hair analysis, accounting for 59.9% of the total forensic toxicology casework; in 2019, 24 873 cases requested hair analysis, accounting for 65.3% of the total forensic toxicology casework. During the period of August 2018 to July 2019, our laboratory received a total of 5 610 cases request for hair analysis, of which approximately 30% (1 713 cases) tested positive for abused substances; that is, the abused substances in hair exceeded the threshold set by the Specifications for the Detection of Drugs in Human Hair Samples and SoHT. Among the posi­tive cases, the age of drug abusers was 15–74 years old, and those under 44 years of age accounted for 77% of drug abusers. Seventy-five percent of positive cases were male drug addicts (Figure 2) [34].

In this study, the most frequently abused substances were amphetamines (56.6%), including methamphe­tamine (MA, 48.0%), amphetamine, methylene dioxymetham-phetamine (MDMA, 8.4%) and methylene dioxyamphetamine (MDA, 0.1%); these substances were used much more frequently than tetrahydrocannabinol (THC, 14.1%), 5-MeO-DIPT (8.1%), cocaine (COC, 7.8%), ketamine (K, 6.8%), and MOR/herion (3.9%), flunitrazepam (2.0%), and others (0.6%).

Decontamination of hair sample

The drugs are incorporated in to hair *via* blood circulation, diffusion from sweat and sebum cutaneum, and external contamination. Different approaches of hair decontamination were applied before hair sample treatment to remove the hair care products, sweat and other surface materials, and avoid false-positives caused by passive exposure to the drug or through environmental exposure. The decontamination solvents include those used to wash the hair such as detergents, sodium dodecylsulfate, aqueous solvents such as water, phosphate buffer, and organic solvents such as ethanol, methanol, isopropanol, diethyl ether, acetone, dichloromethane, pentane, and hexane. The non-polar solvent has the advantage of not expanding the hair, while the polar solvent may promote the dissolution of the binding drugs due to the expansion of the hair. The smaller the polarity of the solvent, the lower the ability of expanding the hair, and the worse the pollution removal effect. There is no common agreement on the washing methods, while it is generally accepted that organic solvent such as acetone will remove only surface contamination and that aqueous solutions or methanol will swell the hair and extract drugs within the hair matrix. In our laboratory, the hair sample was washed with acetone and water [8], in accordance with the recommendation of a washing procedure with both organic solvent and aqueous solutions washing by SoHT [35]. The similar wash steps were also applied by other researchers. For example, the hair samples were washed twice washed with methylene chloride and methanol in sequence [36], or using isopropanol followed by sequential aqueous washes [37], or rinsed with dichloromethane and water in sequence [38].

For damaged or porous hair samples, aggressive and extended aqueous or 90% ethanol washing of hair samples is a proven method for removing or identifying externally derived drug contamination of hair [39]. The amount of drug in the last wash was often used to calculate a wash criterion to determine whether samples were positive due to use or contamination, as well as the metabolite to drug ratio.

### Drug-facilitated crime cases

**Figure 3.**
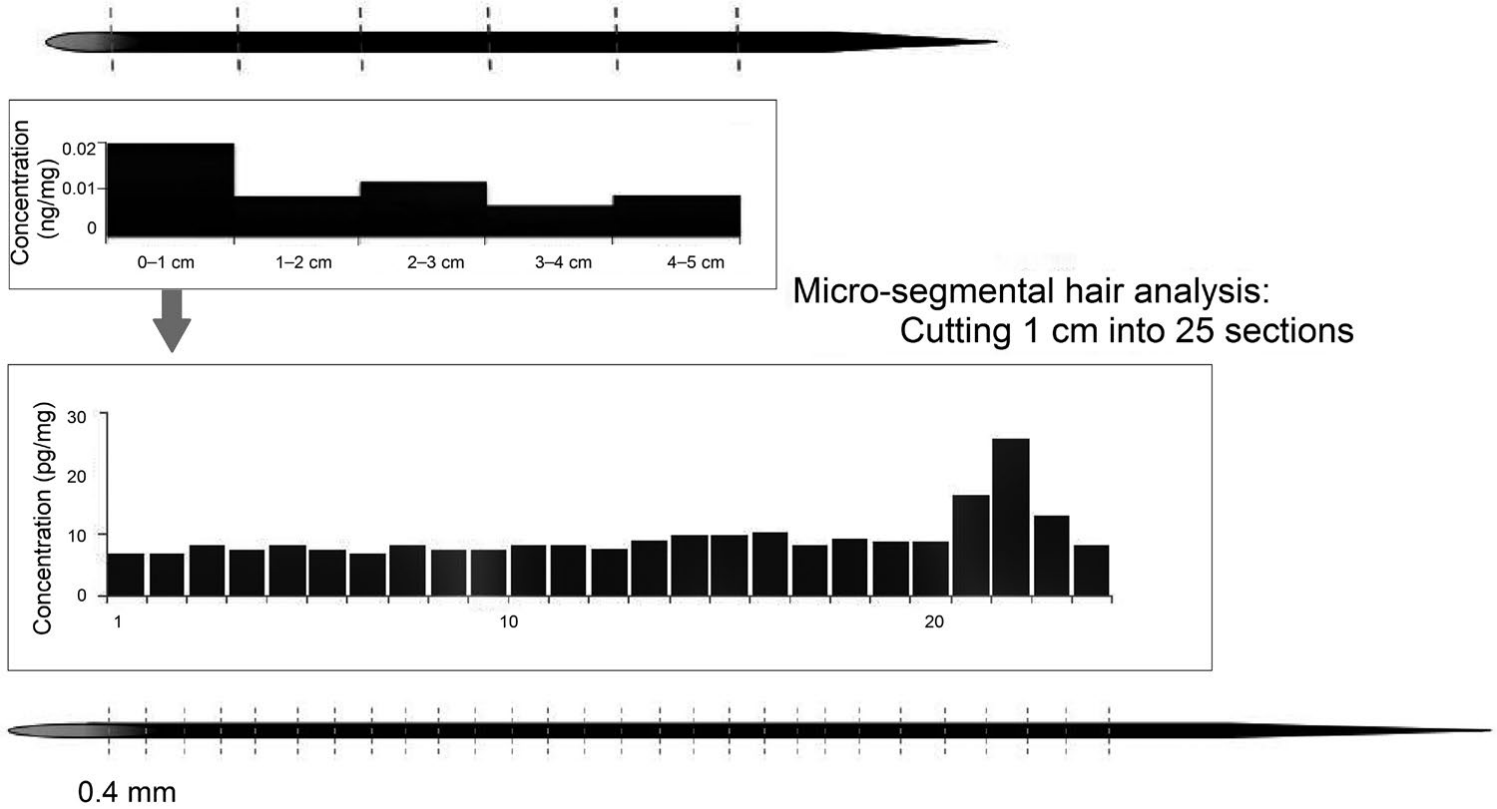
Conventional segmental and micro-segmental results (unpublished observations).

A drug-facilitated crime (DFC) refers to sexual assaults, robbery and other criminal acts facilitated by psychoactive substances. With the development of LC-MS/MS and GC-MS/MS technologies, segmental hair analysis has been applied in documented single dose administration. Drugs were detected in 2–3 consecutive segments in 2011, probably due to the accumulation of benzodiazepines in black hair. In a controlled study [40] of single zolpidem admini­stration (10 mg, *n* = 20), hair sample was collected 1 month after administration and analyzed using LC-MS/MS. All of the proximal 0–2 cm segments were found to contain zolpidem; concentrations ranged from 135.0 to 554.6 pg/mg. Seven 2–4 cm segments tested positive for zolpidem. In another study, the hair of the victims in DFC cases was cut into smaller segments of 0.5 cm to study the distribution of clonazepam and to infer the drug ingestion time (Figure 3) [41]. In one recent study [42] on the kinetics of antipsychotic drugs in hair, hair segments were cut into 3 mm lengths, which helped to find the hair segments with peak concentration and infer the ingestion time. Kintz [43] suggested that the drug intake pattern should be inferred according to the drug concentration in 2-cm hair segments corresponding to the time of the crime and the adjacent hair segments assuming a hair growth rate of approximately 1 cm/month.

Glycosylgotd hemoglorubbisexualsh bisexualn (GHB) is not only a recreational drug abused in drug-facilitated sexual assault (DFSA) cases and other contexts, but is also a therapeutic agent for narcolepsy, as well as an endogenous substance in humans. Hair analysis is a unique way to document GHB exposure when the collection of biological samples occurs more than 8 h after an offence [44]. However, it is difficult to differentiate endogenous and exogenous GHB in hair by quantitative analysis. The ave­rage concentration of endogenous GHB in the hair of a member of the Caucasian population is (0.90±0.37) ng/mg (*n* = 19) [45], whereas the endoge­nous GHB concentration in the hair of a sample of the Chinese population was (1.93 ± 1.40) ng/mg (*n* = 66) [11]. It is recommended to avoid collection of hair over a period longer than 3 months to docu­ment a single GHB exposure to minimize displacement by radial migration of the hair. The analysis of GHB in relatively small hair segments may help achieve positive hair test results in a relatively narrow time frame. The United Nations Office on Drugs and Crime (UNODC) suggests that a strand of hair has to be cut into 5 to 10 small segments (0.3 to 0.5 cm-long) if one segment has a GHB concentration 10 times higher than that of the other segments, suggesting administration of exogenous GHB [46].

### Anti-doping practice

The advantages of hair analysis indicate its potential for applications in the field of anti-doping. Hair analysis can reflect a period of drug intake history of athletes and is expected to replace or supplement the spot test. It can be used as an adjunct to urine analysis to distinguish single and multiple doses and to identify the source of the substance (exogenous or endogenous) and the original form (ester or derivative). Our laboratory has carried out research on the detection and evaluation of anabolic steroids in hair. Shen et al. [15] described a liquid chromato­graphy-­tandem mass spectrometry method for the simultaneous determination of 10 anabolic androgenic steroids and 11 anabolic androgenic steroid esters in hair. The LOD and LOQ ranged between 0.001 and 0.02 ng/mg and between 0.002 and 0.04 ng/mg, respectively. The time courses of the concentrations of anabolic steroids (methyltestosterone, stanozolol, methandienone, nandrolone, trenbolone, boldenone, methenolone and dehydroepiandrosterone (DHEA)) in hair were investigated by our laboratory [47]. It was demonstrated that the peak concentrations were reached on days 2–4, except for stanozolol, which peaked on day 10 after administration. After multiple administrations, anabolic steroids could accumulate in the hair, and the concentrations of anabolic steroids in hair appeared to be related to the physical and chemical properties and dosage of the drug. In addition, the physiological level of endogenous anabolic steroids in hair is important to distinguish between naturally generated steroids and exogenous steroids. Shen et al. [16] studied the physiological concentrations of testosterone, epitestosterone, androsterone, phenylcholinone, and dehydroepian­drosterone in the hair of 80 Chinese people. Table 2 shows that the average concentration of DHEA in hair was much higher than that of testosterone. Men have higher concentrations of testosterone and DHEA in their hair than women, and children have lower concentrations than women.

**Table 2.** Concentrations of endogenous anabolic steroids in the hair of Chinese people. Adapted with permission [16].

	Male (pg/mg) (n=39)			Female (pg/mg) (n=30)			Children (pg/mg) (n=11)		
Component	Medium	Mean	SE	Medium	Mean	SE	Medium	Mean	SE
Testosterone	6.0	7.4	0.9	3.1	5.3	0.9	1.4	2.6	1.0
DHEA	40.2	80.7	16.0	31.7	36.6	3.5	9.5	24.0	9.5
Androsterone	1.3	2.4	0.6	0.7	1.1	0.2	0.3	0.9	0.4
Phenylcholinone	0.6	1.4	0.7	0.4	0.5	0.1	0.3	0.4	0.1
Epitestosterone	1.0	1.2	0.2	1.0	3.4	2.1	0.3	0.3	0.3

Although hair analysis has not been recognized by the World Anti-Doping Agency, it is accepted in courts of justice. In 2015, zearalenone was detected in the hair of an athlete whose urine was positive for selenol and who was suspected of doping. We found that zearalenone could be metabolized into selenol in animal experiments and that zeranol and zearalenone exposure could be discriminated by hair analysis (unpublished observations).

All in all, more research results and case studies are needed before hair analysis can be applied in the field of anti-doping, especially when evaluating drug concentrations in hair, which is related to the level of melanin and may result in racial problems, in addition to the problems caused by a lack of positive quality control samples.

### Postmortem toxicology

Hair analysis is increasingly applied in postmortem toxicology in China. One of its supposed applications is to help identify the characteristics and identities of the dead, such as drug addicts, mental patients, and smokers, to provide clues and directions for cases (unpublished observations). In 2000, police found a female skeletonized corpse in an old empty house. Segmental hair analysis revealed that she had been abusing heroin for a long time; this finding helped the police investigate the drug abusers and finally cracked the case. In 2013, police found the body of a highly corrupt man with his hands and feet bound in a canal, and the legal examiner concluded that he had been dead for more than 30 days. The complexity of the local water network prohibited the body from being identified. Clozapine and its metabolites were detected in a blood clot of the deceased in our laboratory. To determine whether the deceased ingested a single high dose or was a patient who took cloza­pine for a long time, hair and nail samples of the deceased were collected for testing. The distribution of clozapine in the hair and nail indicated a long history of clozapine use [17]. Therefore, it was concluded that the deceased was possibly a psychotic patient who took clozapine for a long time. Then, the police identified the corpse within 24 h. Hair analysis can provide further information to distinguish chronic (prolonged exposure) or acute (single exposure) poisoning if positive drugs test results are acquired in conventional biologic samples.

The authors believe that the potential information contained in hair roots (hair follicles) can expand the application range of hair analysis. Since 2018, we carried out guinea pig experiments with quetiapine and midazolam to study its absorption and elimination in hair roots and blood. Midazolam, quetiapine and their metabolites can be detected in the hair root 5–15 min after administration of a single dose. The drug and metabolite can quickly enter the hair root through the bloodstream. Midazolam can be detected in hair roots 21 days after a single exposure [48], and the detection window of quetiapine can extend to 28 days [18], which means that the drug remains in the hair root longer than it remains in the blood. The study results show that the hair root could provide a supplementary for blood sample in acute poisoning cases due to the extended detection window of weeks, especially when there is a lack of blood samples or drug elimination from blood (e.g. delayed death). In addition, hair root and hair shaft analysis contributes to the development of forensic science. Hair shaft analysis can provide the drug intake history, which can be used to explain pathological autopsy findings, such as myocyte hypertrophy, contraction band necrosis, brain cell necrosis introduced by long-term amphetamine use, and hair root analysis can promptly provide information on drug intake, which can help determine whether death is due to acute poisoning.

Hair root samples from 39 deceased individuals were collected and analyzed, all of which were not caused by drug poisoning (unpublished observations). The qualitative and quantitative results are shown in Table 3. No drugs were detected in hair roots or blood samples from 11 cases. The same drugs were detected in hair roots and blood samples from six cases, which indicated that hair root analysis could provide instant information on drug ingestion. Drugs were detected in hair roots but not in blood in 21 cases, indicating that hair roots had a longer detection window than blood. Indomethacin was detected in blood in only one case but not in hair roots.

**Table 3.** Drug detected in hair root samples from 39 deceased individuals.

Compound	Case number	concentration (ng/mg)
Chlorpheniramine	15	0.024–61
Acetaminophenol	9	1.0–20
Ephdrine	6	0.019–1.3
Amlodipine	4	0.053–0.61
Metoprolol	4	0.47–7.1
Ambroxol	4	0.080–6.0
Lidocaine	4	0.054–22
Tramadol	3	0.50–249
Atracurium	3	0.80–12
Domperidone	2	0.40–0.91
Ondansetron	2	0.040–0.91
Dexamethasone	2	0.33–0.40
Metoclopramide	2	0.033–2.5
Anisodamine	1	2.8
Indomethacin	1	0.35
Aminophenazone	1	2.3
Atropine	1	0.048
Antipyrine	1	2.0
Estazolam	1	0.58
Alprazolam	1	0.011
Cimetidine	1	5.9
Ropivacaine	1	5.6
Cocaine	1	17
Benzoylecogonine	1	0.070

## Further development of hair analysis

Hair analysis has been widely used in the field of forensic science, but it should be noted that it still has some limitations in providing evidence of drug ingestion. First, using hair analysis to infer the pattern of drug use (long-term drug use, single dose use) is based on the time model to clarify whether the subject ingested a drug in a specific period of time or repeatedly used the drug in a specific period of time. However, the estimation of the time and mode of exposure by hair analysis still has a high level of error, with insufficient accuracy and precision, and its application scope and ability to provide conclusive evidence in drug intake time estimation are still restricted. Combining the current research and case work of hair analysis, the authors infer that the time resolution error is due to the indirect, comprehensive and rough information provided by hair analysis technology. The spatial distribution of a drug on a single hair can be reflected by analysis of the bundle consisting of multiple hairs.

The estimation error of drug intake by segmental hair analysis is affected mainly by the growth cycle of hair, drug incorporation mechanism and sampling error. First, a drug is distributed in hair at different distances from the root due to the variation in the growth rate of hairs from the same individual. Hair grows in cyclic periods, including the growing phase (anagen), regressive phase (catagen) and dormant resting phase (telogen). Usually, approximately 80% of the hair on the head is in the growth phase, and 20% is in the regressive or dormant phase. Because the hairs in a bundle are probably in different stages of the growth cycle, a drug introduced in the growing phase of the hair shifts from the root to the end with the growth of the hair; contrastingly, a drug introduced in the resting phase of the hair still stays in the original position of the hair with a lag in displacement, then the inconsistent superposition resulted in the error of time estimation by the segmental analysis of the bundle. Second, there are multiple routes for the drugs to enter into the hair, mainly entering dermal papilla cells through blood circulation during the formation of the hair and through secretion from sweat and sebaceous glands or external pollution attached on the surface of the hair shaft. The different routes of drug binding in hair make the drug widely distributed. Third, a sampling error can also lead to an inaccurate estimation of drug ingestion time. The surface of the head has a certain radius. The distances between the clipping point and the scalp were not equal when a bundle of hair was cut simultaneously from the scalp. Finally, the segment length would limit the accuracy of the estimation of drug ingestion time. Segmental hair analysis with hair segments in centimeters could not effectively capture the peak concentration in hair. All the above factors may lead to a certain diffusion of the peak concentration in hair, which leads to a large deviation in the correspondence with the time of drug intake.

**Figure 4.**
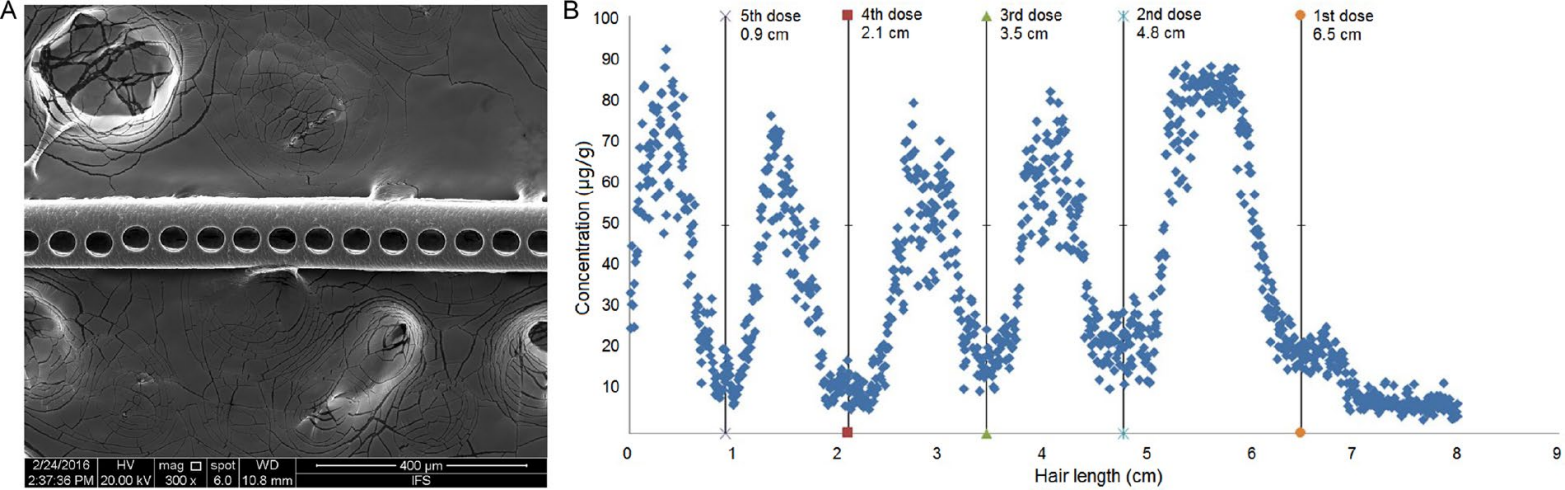
Post-ablation photo of hair sample (A) and arsenic distribution profile in a hair strand (B). Adapted with permission [49].

Microsampling technology based on a single hair may be a good choice for estimating drug intake time. The vertical distribution and peak position of the drug in the hair shaft will be investigated *in situ* by microsegments of a single hair in millimetres or submillimeters. Higher spatial resolutions correspond to higher temporal resolutions, and an accurate ingestion time can be attained. We compared microsegmental hair analysis (hair segments were cut in 0.4 mm pieces) with regular segmental hair analysis (hair segments cut in 1 cm pieces) using hair samples from DFC victims. A single 0.4 mm hair segment was soaked in extraction solvent and the supernatant was transfered and analysed by LC-MS/MS. The drug peak was easily found after microsegmental hair analysis, and the intake time of midazolam was inferred more accurately (Figure 3) (unpublished observations). LA-ICP-MS technology was used to investigate the longitudinal distribution of arsenic in a single hair *in situ*, and a mathematical model was established to predict arsenic entry time based on the retention position of arsenic in the hair and the growth rate of the hair [49] (Figure 4). A method for manually segmenting single hairs in 2-mm sections and screening for 156 analytes using LC-MS/MS was developed, and applied in 15 single dose cases by analyzing ten hairs each [50]. Many substances including mirtazapine could be detected in the presented cases, whereas detection of benzodiazepines and low dosed opioids remains challenging. Therefore, the microsampling technique based on a single hair may be a reasonable way to infer the time of drug ingestion with high accuracy. Obviously, the limitation of a single hair analysis is the small drug amount in it which require high sensitivity of the analytical method.

The evidential value of hair analysis will be enhanced as the entry and retention mechanism of drugs in hair becomes clearer. Scholars worldwide have been exploring methods to study the binding mechanism of drugs in hair and to verify the theoretical hypothesis of whether the binding of drugs into hair can occur in the process of hair formation or can still enter *via* multiple routes after hair forms. The retention of drugs in different layers of hair (surface layer, hair shaft, etc.) can be determined by treating hair with different solvents and methods, but there are large deviations in the results. The distribution of drugs cannot be obtained using microscopic infra-red technology due to the lack of sensitivity and resolution. More efforts are still needed to verify the entry and retention mechanisms of drugs in hair, especially in relation to melanin. The past 30 years of research have mainly focussed on hair shafts, and research on hair roots is rare although hair shafts are an important source of drug information. The drug entry and retention mechanism can be explored if the distribution and kinetics model of drugs in hair roots are investigated.

Furthermore, with the development of imaging mass spectrometry (IMS) technology, it is possible to carry out *in situ* qualitative and quantitative analysis of hair. In 2014, our laboratory determined the longitudinal distribution of ketamine in a single hair of a ketamine abuser [51]. In 2017, Wang et al. [52, 53] developed an MSI method using matrix-assisted laser desorption/ionization-Fourier transform ion cyclotron resonance (MALDI-FTICR) for the direct identification and imaging of methamphetamine and olanzapine in hair samples. MALDI imaging intensities in single hairs showed good semiquantitative correlation with the LC-MS/MS results. In 2020, MALDI-FTICR MSI was developed for direct identification and imaging of synthetic indole-3-carboxamide cannabinoids in hair samples [54]. Recently, we used TOF-SIMS to investigate the radial cross-sectional distribution and binding mechanism of ketamine in the hair of ketamine abusers. IMS has gained attention from scientists in Japan, Switzerland, Belgium and the Netherlands [55–57].

In conclusion, the estimation of drug intake time can be more accurate if the spatial distributions of drugs in hair shafts are characterized at a higher resolution. A single hair analysis *in situ* would make the peak band of the drug narrower, reduce the hair growth variation and minimize sampling error. The entry and incorporation of drugs into hair will be shown more clearly, and the results of hair analysis can be elaborated to reveal the drug use history specific by days.

The evidential value of the hair matrix was promoted due to the efforts of the researchers, and its evidentiary power has been recognized internationally. In 2020, more than 200 laboratories participated in proficiency testing programmes for hair analysis of abused drugs in China. The special target compounds in cases and the accumulation of drugs in black hair enabled hair analysis of regional characteristics. Nevertheless, the mechanism of drug binding in hair, the quality control of hair analysis and the interpretation of hair analysis results still need to be considered.

## Authors’ contributions

Hui Yan researched the topics, reviewed literature, and wrote the initial rough draft. Ping Xiang reworked the Introduction and edited the draft. Min Shen established an outline, and edited the overall flow of the paper’s focus and perspective. All three authors contributed to and revised the final version of the manuscript.
